# Association of Antioxidant Diet with Risk of Hyperemesis Gravidarum Among Chinese Pregnant Women: A Population-Based Cross-Sectional Study

**DOI:** 10.3390/nu17030598

**Published:** 2025-02-06

**Authors:** Lan Zhang, Xiang Li, Yuan Jin, Wenjie Cheng, Xinyu Zhang, Qian Ma, Aohua Liu, Siyang Chen, Yahui Fan, Shunming Zhang, Jing Lin, Le Ma

**Affiliations:** 1 School of Public Health, Xi’an Jiaotong University Health Science Center, Xi’an 710061, China; zhanglan@stu.xjtu.edu.cn (L.Z.); 18835427692@163.com (X.L.); jinyuan0828@stu.xjtu.edu.cn (Y.J.); chengwj20@163.com (W.C.); zhangxinyuzc@163.com (X.Z.); maqian9871@163.com (Q.M.); liuaohua@stu.xjtu.edu.cn (A.L.); chensiy@stu.xjtu.edu.cn (S.C.); fyh14042166@stu.xjtu.edu.cn (Y.F.); 2 Key Laboratory for Disease Prevention and Control and Health Promotion of Shaanxi Province, Xi’an Jiaotong University, Xi’an 710061, China

**Keywords:** composite dietary antioxidant index, dietary antioxidant potential score, reduced rank regression, oxidative stress, hyperemesis gravidarum

## Abstract

(1) Background: Oxidative stress plays a pivotal role in the pathophysiologic of hyperemesis gravidarum (HG). Epidemiological studies have explored the associations of specific antioxidant foods and nutrients with HG. However, evidence regarding the relationship between an antioxidant-rich diet and the risk of HG remains limited. The objective of this research was to explore the relationship between antioxidant-rich diet and HG. (2) Methods: This was a population-based cross-sectional study. A total of 2980 pregnant women were included in our population. A composite dietary antioxidant index (CDAI) was calculated by summing the standardized intakes of vitamins A, C, and E, selenium, zinc, and total carotene. A dietary antioxidant potential score (DAPS) was derived using reduced rank regression. Binary logistic regression models were employed to analyze the associations of CDAI and DAPS with risk of HG. (3) Results: In total, 241 (8.09%) cases of HG were identified in this study. After adjusting for potential confounders, including age, socioeconomic status, ethnicity, physical activity, current smoking status, current alcohol consumption, pre-pregnancy body mass index, nutritional supplement usage, total energy intake, gestational week, menstruation regularity, family history of HG, primigravida status, and quality of life during pregnancy, ORs (95% CIs) of HG in the highest tertiles were 0.31 (0.21–0.47) for CDAI and 0.41 (0.28–0.57) for DAPS when comparing lowest tertiles (all *p*-trend < 0.001). Such associations remained robust across multiple sensitivity analyses and subgroup analyses. (4) Conclusions: Higher CDAI and DAPS, indicative of greater adherence to an antioxidant-rich diet, were associated with a lower risk of HG. This finding underscores the crucial role of consuming antioxidant-rich foods in the prevention of HG.

## 1. Introduction

Hyperemesis gravidarum (HG) is a prevalent pregnancy complication, characterized by severe nausea and vomiting (NVP), which can lead to significant weight loss, dehydration, electrolyte imbalances, ketosis, and even Wernicke encephalopathy. HG affects approximately 14% of pregnancies worldwide [1]. The incidence rate of HG varies from 0.3% in Europe and 10.8% in China [2]. HG poses increased risks to both the short- and long-term health of mothers and their offspring, including anxiety disorders, preeclampsia, eclampsia, post-traumatic stress disorder [3], preterm birth, hyperactivity disorder, and autism spectrum disorder [4,5]. However, current treatments targeted for HG (e.g., medication, and parenteral nutritional supplementation, etc.) have not been shown to effectively reduce the risk of adverse health outcomes in offspring. Therefore, identifying potentially modifiable risk factors may play a crucial role in preventing HG.

Oxidative stress is a critical factor in the pathophysiologic of HG [6]. In particular, elevated mitochondrial activity in the placenta increases the production of reactive oxygen species (ROS) [7]. This disruption of oxidation balance during early pregnancy contributes to the onset of HG [6]. Randomized clinical trials have demonstrated that dietary interventions, such as the addition of watermelon and ginger capsules (possessing antioxidant properties, 500 mg twice daily), can effectively alleviate HG symptoms [8,9,10]. Furthermore, a nested case–control study suggested that dietary intake of vitamins A, C, E, and carotene (with notable antioxidant properties) may help reduce the incidence of HG in pregnant women [11]. Moreover, our previous studies indicated an inverse association between dietary patterns rich in antioxidant foods, including fish, shrimp, egg, milk, and water, and the risk of HG [12]. Similarly, our prior cross-sectional study evaluating the dietary inflammatory levels of pregnant women found an inverse relationship between individual vitamins C, D, and E, zinc, selenium, and HG [13]. However, individual foods and nutrients might not represent an individual’s overall antioxidant intake [14]. In this regard, Wright et al. developed a composite dietary antioxidant index (CDAI) to estimate an individual’s overall antioxidant levels [15,16]. In addition, reduced rank regression (RRR), serving as one of the tools for constructing dietary patterns, utilizes prior knowledge to identify food groups associated with specific diseases in a given population [17]. Compared to other methodologies, the dietary patterns based on RRR exhibit a more robust association with specific diseases. The impact of these dietary patterns on disease risk can be described through changes in biologically significant intermediate variables, thereby facilitating a more thorough examination of the effects of dietary patterns on the disease [17,18,19,20,21].

However, to the best of our understanding, the association antioxidant-rich diet based on CDAI and RRR with HG was poorly understood. Thus, we conducted this population-based study to examine the relationship between CDAI, DAPS, and HG risk among Chinese pregnant women. Thus, we hypothesized a diet rich in antioxidants associated with a reduced risk of HG.

## 2. Materials and Methods

### 2.1. Study Population

This study utilized data derived from the Xi’an Birth Cohort Study (XBC), an auxiliary component of the China Birth Cohort Study, which is a population-based cohort study initiated in 2017. The principal aim of the present research endeavor is to elucidate the association between maternal nutrition, lifestyle, and environmental factors, as well as their impacts on maternal and offspring health, with the overarching goal of mitigating adverse perinatal outcomes morbidity in China [13,22]. Comprehensive details regarding the study design and profile have been documented in our previous publications [13]. Briefly, the cohort comprised pregnant women, aged from 18 to 49 years, who were recruited during their initial antenatal examination at 6 to 14 weeks of gestation. Data on sociodemographic characteristics, anthropometric measures, and lifestyle factors were gathered through structured electronic and self-administered questionnaires at the time of enrollment, while obstetric information was extracted from medical records. The current analysis focused on participants from the third phase of the XBC, which spanned from April 2021 to September 2023. The research conducted adhered to the ethical guidelines established in the Helsinki Declaration, with informed written consent obtained from all participants, and ethical approval granted by the Medical Ethics Committee of the Xi’an Jiaotong University Health Science Center (No.2020–1263).

**Figure A1:**
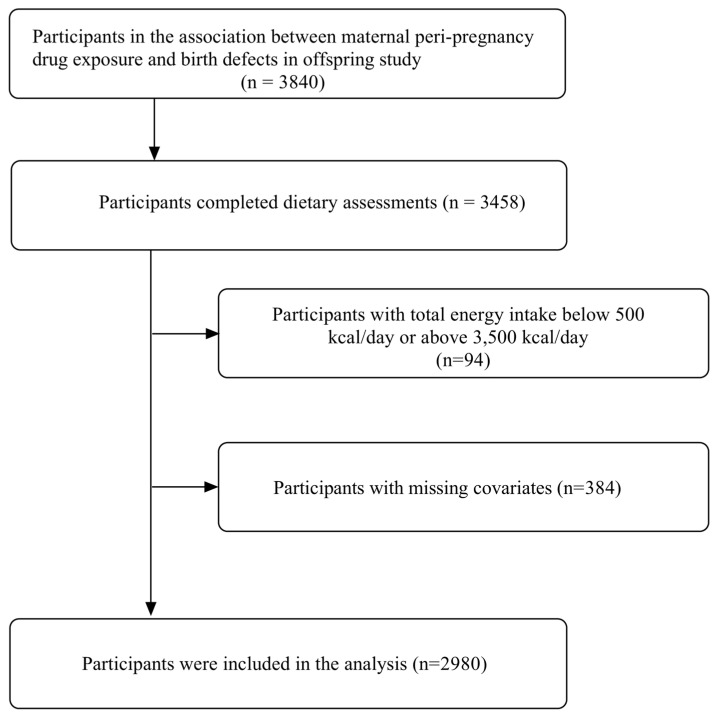
The flow diagram of participant selection.

In this cross-sectional study, 3480 individuals were recruited, with 3362 completing the dietary assessment. Participants with implausible total energy intake (<500 or >3500 kcal/day) (*n* = 94) and those with missing covariates data (*n* = 384) were excluded. Consequently, 2980 individuals were incorporated into the ultimate analysis (Figure A1).

### 2.2. Dietary Assessment

Dietary consumption information was gathered utilizing a validated, semiquantitative food frequency questionnaire (FFQ), comprising 10 major categories and 108 items [23,24]. A food atlas, featuring nine levels of consumption frequency, was employed to assist participants in accurately completing the FFQ. Participants were instructed to recall and report their usual consumption frequencies of individual food items over the past year. The method for calculating total energy intake and other nutrient content adhered to the procedures delineated by Fan et al. [22].

### 2.3. Construction of CDAI and DAPS

Consistent with previous studies [16,25], the CDAI was derived from the dietary intake of six vitamins and minerals, including vitamins A, C, and E, zinc, selenium, and total carotenoids, excluding contributions from supplements and medications. Each nutrient was normalized by subtracting the global mean and dividing by the standard deviation, and the CDAI was computed as the aggregate of these normalized intakes, each weighted equally. Furthermore, DAPS was derived by RRR [18,26]. In RRR, the food items were classified into 26 categories, with categorization based on similar culinary techniques or nutrient profiles. To elucidate the primary factors contributing to the DAPS, the initial factor from RRR was retained as the dependent variable in subsequent stepwise linear regression analysis, incorporating the 26 food groups as independent variables [18]. Food groups retained in the final model were determined based on a significance threshold of *p* = 0.2 (Table A1). Notably, pro-oxidant foods included edible animal oils, processed meat, livestock and poultry products, rice, and beverages, while antioxidant foods encompassed whole grains, mixed legumes, fruits, eggs, fish, dark green vegetables, white leafy vegetables, yogurt, milk, and water. Ultimately, participants’ DAPS was calculated by weighing the intake of each food component according to the regression coefficients in the stepwise linear regression model. Higher CDAI and DAPS scores reflect a greater consumption of antioxidant-rich foods.

### 2.4. Ascertainment of HG

HG was diagnosed when participants exhibited any of the following criteria: Pregnancy-Unique Quantification of Emesis and Nausea (PUQE) scores ≥13 [27]; significant nausea and vomiting interfered daily activities, including normal eating and drinking, alongside visible signs of dehydration [28]; weight loss exceeding 5% of pre-pregnancy weight due to nausea and vomiting prior 16 weeks of gestation [29]; or hospitalization for comprehensive therapeutic interventions for HG [30]. The diagnosis was confirmed through medical records and a self-administration questionnaire.

### 2.5. Assessment of Covariates

**Figure A2:**
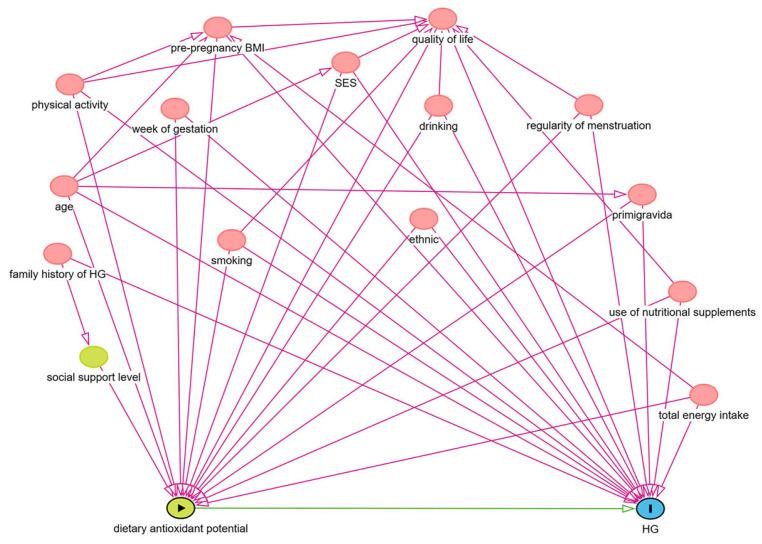
Directed acyclic graph showing independence among potential confounding variables on the association between the dietary antioxidant potential score and hyperemesis gravidarum risk. The solid rose-pink circles are indicative of common ancestral factors shared by both the exposure and the outcome. The solid light green circle, marked with black triangles, represents exposure factor. Meanwhile, the solid light blue circle, adorned with black rectangles, signifies outcome factor. The solid light green circle, devoid of any additional marks, represents ancestral factors related to exposure. Dark green arrow is utilized to depict causal pathways, whereas rose-pink arrows are employed to illustrate bias pathways. Confounding factors included age, pre-pregnancy BMI, nutritional supplement usage, and total energy intake, the gestational week, SES, ethnicity, menstruation regularity, family history of HG, quality of life during pregnancy, physical activity, primigravida status, current smoking status, and current alcohol consumption. Abbreviations: BMI: body mass index; HG: hyperemesis gravidarum; SES: socioeconomic status (including employment status, annual household income, and educational attainment).

Directed acyclic graph (DAG) was employed to identify and select potential confounders and integrate evidence synthesis methodologies with causal inference principles to enhance the rigor of confounding factor assessment [31,32]. Sociodemographic information (age, gestational weeks, employment status, educational attainment, annual household income, and ethnicity), lifestyle habits (smoking status, alcohol consumption, physical activity, quality of life, pre-pregnancy body mass index (BMI), and nutritional supplements usage) and obstetrics history (menstruation regularity, family history of HG, and parity) was collected via self-administered questionnaires. Occupational status was dichotomized as either employed or unemployed, while educational attainment was classified as under college or college and above. Annual household income was stratified into categories of < CNY 100,000 and ≥ CNY 100,000 and ethnicity was classified as Han or minority. Other variables were dichotomized as yes or no, including smoking status, alcohol consumption, nutritional supplement usage, menstrual regularity, and family history of HG. Parity was categorized as primigravida or multipara. BMI was computed as the ratio of weight in kilograms (kg) to the square of height in meters (m^2^). The level of physical activity was evaluated through the utilization of the International Physical Activity Questionnaire (IPAQ) Short Form, which calculates the average daily metabolic equivalent (MET-min/week) based on the frequency and duration of various activity levels. Quality of life was evaluated by the PUQE scale. Based on causal diagrams and established criteria, we identified age, gestational weeks, employment status, educational attainment, annual household income, ethnicity, parity, pre-pregnancy BMI, physical activity, quality of life, menstruation regularity, family history of HG, smoking, alcohol consumption, total energy intake, and nutritional supplement usage as confounding variables (Figure A2).

### 2.6. Statistical Analysis

Participants’ characteristics were described as means ± standard deviation (SD) for normally distributed variables, medians (interquartile range) for skewed distributions, and counts (proportions) for categorical data. To evaluate the association between the CDAI and DAPS with HG, binary logistic regression analyses were employed to ascertain odds ratios (ORs) and their corresponding 95% confidence intervals (CIs). The initial model (Model 1) was unadjusted for any confounding variables. Model 2 incorporated adjustments for age, pre-pregnancy BMI, nutritional supplement usage, and total energy intake. Model 3 further adjusted for gestational week, educational attainment, employment status, ethnicity, menstrual regularity, family history of HG, quality of life during pregnancy, physical activity levels, parity, annual household income, smoking status, and alcohol consumption. The variance inflation factor (VIF) was calculated to assess multicollinearity among nutrient intake variables, with all VIF values below 1.5, indicating an acceptable degree of collinearity in model 3 [33]. The relationships among six distinct nutrient levels were analyzed using Spearman’s nonparametric rank-order correlation due to the observed skewness in their distributions. Additionally, *P* for trend was calculated by using median values of CDAI or DAPS tertiles as continuous variables, facilitating the assessment of linear trends in ORs. Restricted cubic spline regression (RCS) with three knots at the 25th, 50th, and 75th percentiles of exposure was utilized to investigate potential dose–response relationships between CDAI, DAPS, and HG.

Several sensitivity analyses were conducted to ensure robustness and explore potential variations in the findings. Initially, we conducted a subgroup analysis, stratified by age (<35, ≥35 years), gestational weeks (<12, ≥12 weeks), primigravida status (yes, no), educational attainment (below college, college, and higher), employment status (yes, no), annual household income (<CNY 100,000, ≥CNY 100,000), pre-pregnancy BMI (<18.5, 18.5~24, ≥24 kg/m^2^), family history of HG (yes, no), current alcohol consumption (yes, no), nutritional supplement usage (yes, no), menstrual regularity (yes, no), physical activity (<median level, ≥median level), quality of life (<median level, ≥median level), and total energy intake (<median level, ≥median level). Furthermore, interactions between CDAI, DAPS, and these covariates were assessed through models both with and without a multiplicative interaction term in a multivariate-adjusted model. The potential confounding effects of total energy intake on disease outcome, as evidenced in previous nutritional epidemiology studies [34,35], new models were fitted to estimate the association between residual energy intake-adjusted CDAI and DAPS with HG. The impacts of a one-SD increment in both the CDAI and DAPS were evaluated when modeled as continuous variables. Additionally, analyses were restricted to participants with complete data, including implausible total energy intake estimates below 500 kcal/day or above 3500 kcal/day. Finally, multiple imputation methods were employed to account for missing covariates [36], and new multivariable logistic models were fitted to estimate the associations between CDAI and DAPS with HG among participants with complete data (excluding implausible total energy intake) and those with missing values, utilizing the multiple imputation techniques.

All statistical analyses were performed using R (version 4.3.2; R Development Core Team), with a two-tailed *p* value of <0.5 considered statistically significant.

## 3. Results

### 3.1. Participant Characteristics

**Figure A3:**
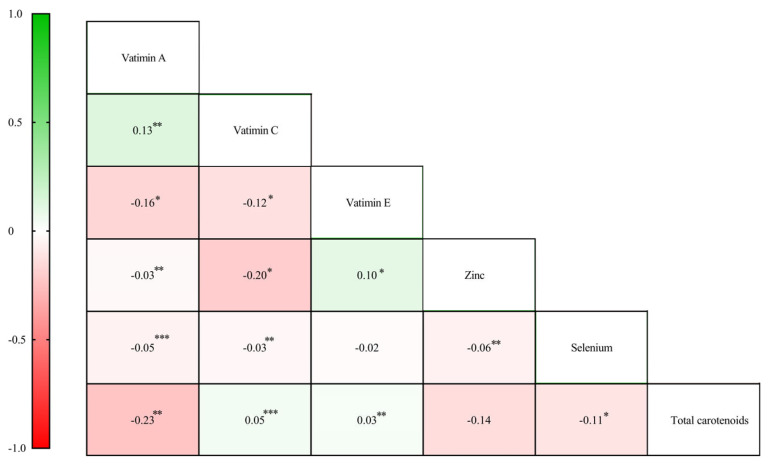
Spearman correlation coefficients across the six nutrients (vitamin A, vitamin C, vitamin E, zinc, selenium, and total carotenoids). In the lower left-hand corner, the strength of each correlation is depicted as color, with red color indicating positive correlation and green color indicating inverse correlation. The darker the color suggests stronger correlations. The Spearman correlation coefficients are shown in the rectangle. The * indicated *p* < 0.05, ** *p* < 0.001, and *** *p* < 0.0001.

In this study, 2980 pregnant women were enrolled, among whom 241 cases of HG were identified, yielding a prevalence rate of 8.09%. Table 1 shows the general characteristics of the participants, both overall and stratified by tertiles of the CDAI and DAPS. The median (25th, 75th percentiles) ages, pre-pregnancy BMI, and gestational weeks were 30.0 (28.0, 33.0) years, 21.2 (19.5, 23.3) kg/m^2^, and 12.0 (9.2, 12.7) weeks, respectively. Additionally, Table A2 presents the general characteristics, food group consumption, intake of six nutrients, CDAI, and DAPS of participants categorized by HG. Figure A3 displays the Spearman correlations among vitamin A, vitamin C, vitamin E, zinc, selenium, and total carotenoids.

### 3.2. Association Between the CDAI and DAPS and HG

**Figure 1:**
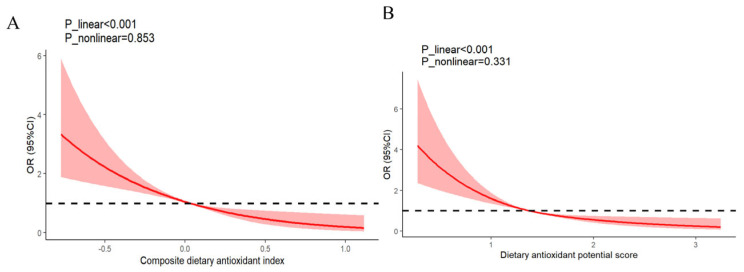
The dose–response relationships of the CDAI (**A**) and DAPS (**B**) with HG. The solid red line describes ORs, and the rose-pink areas show 95% CIs. The logistic models were adjusted for age, pre-pregnancy BMI, nutritional supplement usage, and total energy intake, the gestational week, educational attainment, employment status, ethnicity, menstruation regularity, family history of HG, quality of life during pregnancy, physical activity, primigravida status, annual household income, current smoking status, and current alcohol consumption. Three knots placed the 25th, 50th, and 75th of the exposures. Abbreviations: BMI: body mass index; CDAI: composite dietary antioxidant index; CI: confidence interval; DAPS: dietary antioxidant potential score; HG: hyperemesis gravidarum; OR: odds ratio.

Table 2 presents the relationships between CDAI, DAPS, and HG. In the initial model (Model 1), the ORs (95% CIs) of HG, when comparing the highest tertiles to the lowest tertiles of CDAI and DAPS, were found to be 0.29 (0.20, 0.42), and 0.37 (0.26, 0.52), respectively, with a *p* for trend <0.001. After adjusting for confounding variables including age, pre-pregnancy BMI, nutritional supplement usage, and total energy intake, these associations were attenuated, yielding an OR of 0.31 (95%CI: 0.21–0.47, *p*-trend < 0.001) for CDAI, and an OR of 0.40 (95%CI: 0.28–0.57, *p*-trend < 0.001) for DAPS. Further adjusting for the gestational week, educational attainment, employment status, ethnicity, menstruation regularity, family history of HG, quality of life during pregnancy, current smoking status, and current alcohol consumption, physical activity, primigravida status, annual household income, Model 3 revealed that the relationships between CDAI and DAPS with HG remained. The overall effect estimates for HG, comparing the top tertiles with the bottom tertiles were 0.32 for CDAI (95%CI: 0.21–0.47; *p*-trend < 0.001), and 0.41 for DAPS (95%CI: 0.30–0.60; *p*-trend < 0.001). Figure 1 illustrates the linear relationship between CDAI (A) and DAPS (B) with HG (CDAI: *p* of for linearity < 0.001, and non-linearity: 0.853; DAPS: *p* of for linearity < 0.001, and *p* of for non-linearity: 0.331).

### 3.3. Sensitivity Analysis

**Figure 2:**
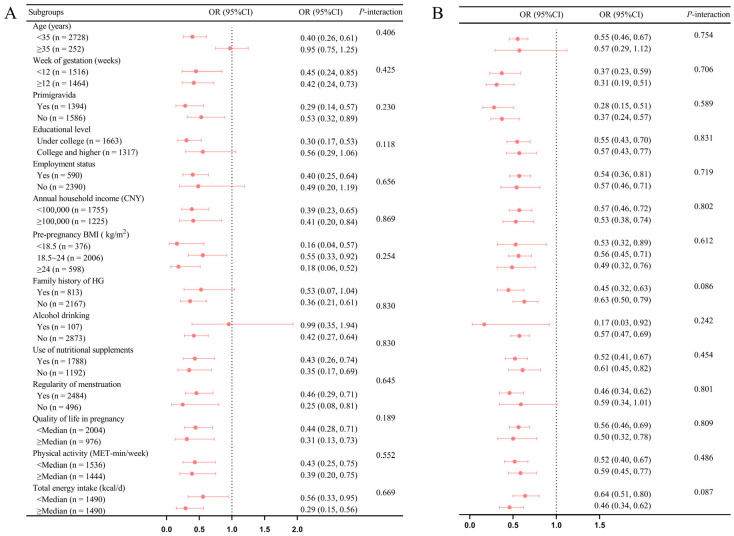
Subgroup analyses for the association of the CDAI (**A**) and DAPS (**B**) with the risk of HG. We applied the composite dietary antioxidant index as a continuous variable (per one SD increase) in the subgroup analysis because of the linear relationships between CDAI and DAPS and hyperemesis gravidarum risk. Subgroup analyses defined by ethnicity and current smoking status were not conducted due to the very small sample sizes. Logistic models were adjusted for age, pre-pregnancy BMI, nutritional supplement usage, and total energy intake, the gestational week, educational attainment, employment status, ethnicity, menstruation regularity, family history of HG, quality of life during pregnancy, physical activity, primigravida status, annual household income, current smoking status, and current alcohol consumption. *P* for interaction was computed by multiplying CDAI and DAPS with this key baseline information into the final model. Abbreviations: BMI: body mass index; CDAI: composite dietary antioxidant index; CI: confidence interval; DAPS: dietary antioxidant potential score; HG: hyperemesis gravidarum; OR: odds ratio.

In the subgroups analysis, no significant interaction was identified between CDAI, DAPS, and various factors including age, gestational weeks, primigravida status, educational attainment, employment status, annual household income, pre-pregnancy BMI, family history of HG, current alcohol consumption, nutritional supplements usage, menstrual regularity, physical activity, quality of life, and total energy intake (all *p*-interaction ≥0.05, as depicted in Figure 2). Furthermore, taking into account the potential confounding effect of total energy intake, the inverse relationships between the residual energy intake-adjusted CDAI (OR: 0.34; 95%CI: 0.23–0.50, *p*-trend < 0.001), DAPS (OR: 0.46; 95%CI: 0.32–0.66, *p*-trend < 0.001), and HG were maintained (Table A3). This consistency was evident when the CDAI (OR: 0.74; 95%CI: 0.64–0.85; *p*-trend < 0.001) and DAPS (OR: 0.56; 95%CI: 0.46–0.67; *p*-trend < 0.001) was treated as a standardized continuous variable within the model (Table 2). In addition, similar inverse associations were identified among all participants including implausible total energy intake estimates, with those consuming less than 500 kcal or exceeding 3500 kcal (OR for CDAI: 0.31; 95%CI: 0.21–0.46; *p*-trend < 0.001; OR for DAPS: 0.41; 95%CI: 0.28–0.59; *p*-trend < 0.001). Finally, the implementation of multiple imputation methods to address missing covariates revealed minor variance in the estimated associations between the CDAI, DAPS, and HG (Table A4).

## 4. Discussion

In this population-based study nested within the XBC, we explored the association between an antioxidant-rich diet and the risk of HG. Our results suggested that elevated levels of CDAI and DAPS, indicating an increased intake of dietary antioxidant ingredients, are associated with a decreased risk of HG. These findings underscore the potential protective role of dietary antioxidants in health outcomes.

To our knowledge, our study was the first to explore the comprehensive joint associations of dietary antioxidant intake and dietary antioxidant potential with the risk of HG. Several previous studies have reported that antioxidant-rich foods, independent vitamins and minerals are linked to HG. Consistent with our findings, Tan et al. reported that pregnant women receiving dietary advice leaflets alongside watermelon supplementation exhibited a notable 26.6% decrease in the risk of weight loss after two weeks, compared to a control group that received only dietary advice. Moreover, the prevalence of severe nausea and vomiting symptoms decreased significantly from 4.7% to 1.6%, contributing to an overall improvement in the well-being of these pregnant women [9]. In addition, Sharifzadeh et al. found that ginger capsules (500 mg twice daily) were more effective than both a vitamin B6 group and a placebo group in reducing the frequency of nausea and retching, as well as the intensity of vomiting. This heightened effectiveness is likely attributed to gingerol, a natural antioxidant found in ginger, which mitigates peroxidation of phospholipids, reduces ROS generation, and consequently lowers the risk of NVP in pregnancy [10,37,38]. Our previous research speculated that dietary sources rich in vitamins C, E, zinc, and selenium may play a protective effect by neutralizing free radicals and reducing oxidative stress in HG [13]. Moreover, in a nest case–control study, Celik et al. demonstrated that dietary levels of vitamin E, vitamin A, vitamin C, carotene, and other antioxidants could reduce the incidence of HG by modulating ROS formation and preventing cellular damage [11]. However, previous epidemiological studies have predominantly focused on the relationships between individual nutrient intake and dietary patterns with HG, leaving the joint association of an antioxidant diet with HG risk less explored.

In this study, the CDAI was calculated by summing the standard intake of six key antioxidant nutrients, while the RRR method was used to derive the DAPS, with CDAI serving as the response variable. Our findings reveal a significant association between elevated CDAI and DAPS and a reduced risk of HG, a relationship that remained robust across multiple sensitivity analyses. This study addresses a notable gap in the existing literature and contributes novel insights into the underlying mechanisms associated with HG and offers a dietary recommendation aimed at its prevention.

However, the underlying mechanism by which adherence to antioxidant-rich diet influences the occurrence of HG remains inadequately understood. Proposed biological pathways suggest that dietary antioxidants may mitigate the risk of HG through several mechanisms, including the reduction in oxidative stress and systemic inflammation. Firstly, HG is recognized as a manifestation of oxidative stress [6]. During early pregnancy, increased metabolic activity within placenta mitochondria leads to an increasing level of ROS [39]. As the intrauterine placental circulation becomes established, the abnormal elevation in placental ROS and superoxide levels induces systemic oxidative stress and precipitating the onset of HG [40]. Evidence suggests that dietary antioxidants can change the expression patterns of pivotal transcription factors implicated in the regulation of mitochondrial biogenesis and ROS generation [41], as well as the capacity to eliminate lipid-based radicals and terminate oxidative chain propagation reactions [42]. Using New Zealand white female rabbits, Sikiru et al. reported that supplementation with antioxidant-rich supplements led to enhanced antioxidant enzyme activity and increased concentrations of glutathione peroxidase, aiding in the inhibition of malondialdehyde production within the body, thereby ameliorating reproductive oxidative stress damage [43]. Another mechanism of association between antioxidant diets and HG may involve reducing inflammation levels within the body. During pregnancy, pro-inflammatory factors activate trophoblastic cells, leading to the release of human chorionic gonadotropin (hCG) [44], and stimulate aromatase in human trophoblast cells, promoting estrogen synthesis [45]. Subsequently, elevated levels of hormone slow intestinal transit time and gastric emptying [46], leading to the occurrence of nausea and vomiting. Research indicated that antioxidant diets exert anti-inflammatory properties by inhibiting the production of pro-inflammatory factors such as interleukin-6 (IL-6), interleukin-1beta (IL-1β), and tumor necrosis factor-alpha (TNF-α) [47,48]. Following intervention with antioxidant-rich diets in rats, Novoselova et al. observed significant reductions in TNF-α, IL-1α, and IL-1β levels in cells and plasma [49]. Plunkett et al. found that compared to the intake of antioxidant-rich diets, dietary antioxidant restriction led to a significant increase in TNF-α concentrations in individuals [50]. In addition, Vahid et al. discovered an inverse correlation between the intake of antioxidant components and serum malondialdehyde and IL-6 in the body [51]. Therefore, it is hypothesized that a diet rich in antioxidant components may suppress inflammatory levels in pregnant women, thereby stabilizing fluctuations in hCG and estrogen levels and reducing the risk of HG. However, it is crucial to acknowledge that excessive consumption of specific vitamins, particularly vitamin A and vitamin D, can result in toxicity, potentially leading to detrimental effects on both maternal and fetal health [52,53]. Further research is warranted to elucidate these mechanisms and their potential implications for dietary recommendations in pregnant individuals at risk for HG.

To our knowledge, it is the first large-scale investigation linking the overall antioxidant diet to HG. In this study, we assess the dietary antioxidant capacity of pregnant women using both antioxidant biomarkers derived from nutrients and antioxidant potential scores derived from foods, and the results were robust across various sensitivity analyses and subgroup analyses. As a hybrid methodology, RRR integrates prior knowledge of nutrients associated with specific diseases with data to derive food-based dietary patterns. This approach allows for the adoption of a “whole diet” perspective, reducing the confounding factors often encountered in studies focusing on single nutrients or foods, while simultaneously considering potential mechanistic aspects of the response variables. Our research provides clinicians with evidence based on foods rather than nutrients, thereby fostering food-based recommendations [17,19,21]. Nonetheless, the study has several limitations. First, the assessment of dietary intake was based on the FFQ, which could potentially introduce inaccuracies in the categorization of dietary intake due to recall bias. However, previous research has demonstrated the relative stability of pre-pregnancy dietary habits over an extended period, suggesting that these habits can reflect dietary patterns throughout pregnancy [54]. Second, the evaluation focused on the intra-pregnancy antioxidant nutrient levels, excluding a range of other antioxidant minerals and nutrients. Nevertheless, existing studies have indicated that the CDAI based on vitamin A, vitamin C, vitamin E, selenium, zinc, and total carotene can effectively assess overall antioxidant levels [15]. Third, food preparation and cooking methods could influence the association between dietary antioxidant levels and HG by affecting the nutritional value and nutrient content of the food [22]. Fifth, due to the observational nature of the design, we cannot establish causal relationships or exclude the impact of potential confounders, despite controlling for numerous confounding variables. Last, the study population primarily comprises Chinese individuals, potentially limiting the generalizability of the findings to other populations. Future research is encouraged to expand the sample size and conduct multicenter prospective studies to confirm our findings.

## 5. Conclusions

In summary, this study identifies an association between higher levels of antioxidant diet intake and a reduced risk of HG. This finding provides new epidemiological evidence and perspectives regarding the dietary protection of HG, suggesting that adherence to an antioxidant-rich diet may be beneficial in preventing HG. Additional investigative efforts are warranted to validate our observations and further elucidate the intricate mechanisms underlying the phenomena.

## Figures and Tables

**Table 1:** General characteristics of participants, total and stratified by tertiles of CDAI and DAPS (*n* = 2980).

Characteristics	Total	Tertiles of CDAI			Tertiles of DAPS		
		Tertile 1	Tertile 2	Tertile 3	Tertile 1	Tertile 2	Tertile 3
Number of participants	2980	993	993	994	994	993	993
Age (years)	30.0 (28.0, 33.0)	30.0 (28.0, 33.0)	30.0 (28.0, 33.0)	30.0 (28.0, 32.0)	30.0 (28.0, 33.0)	30.0 (28.0, 32.0)	30.0 (28.0, 32.0)
Gestational week (weeks)	12.0 (9.2, 12.7)	12.2 (9.3, 12.7)	12.0 (8.8, 12.7)	12.0 (9.3, 12.7)	12.2 (9.2, 12.7)	12.0 (9.0, 12.7)	12.0 (9.3, 12.7)
Educational attainment (under college)	1663 (55.8)	542 (54.6)	554 (55.8)	567 (57.0)	560 (56.3)	541 (54.5)	562 (56.6)
Employment status (no)	590 (19.8)	204 (20.5)	207 (20.8)	179 (18.0)	195 (19.6)	207 (20.8)	188 (18.9)
Annual household income (<CNY 100,000)	1755 (58.9)	606 (61.0)	595 (59.9)	554 (55.7)	601 (60.5)	586 (59.0)	568 (57.2)
Ethnicity (Han)	2928 (98.3)	980 (98.7)	968 (97.5)	980 (98.6)	968 (97.4)	979 (98.6)	981 (98.8)
Primigravida (yes)	1394 (46.8)	447 (45.0)	453 (45.6)	494 (49.7)	485 (48.8)	438 (44.1)	471 (47.4)
Pre-pregnancy BMI (kg/m^2^)	21.2 (19.5, 23.3)	21.0 (19.4, 23.4)	21.3 (19.5, 23.2)	21.2 (19.6, 23.3)	21.1 (19.3, 23.4)	21.2 (19.5, 23.2)	21.2 (19.6, 23.4)
Quality of life in pregnancy	8.0 (7.0, 9.0)	8.0 (7.0, 9.0)	8.0 (7.0, 9.0)	8.0 (7.0, 9.0)	8.0 (7.0, 9.0)	8.0 (7.0, 9.0)	8.0 (7.0, 9.0)
Physical activity (MET-min/week)	1188 (720, 1980)	1188 (726, 1980)	1188 (666, 1980)	1188 (780, 1980)	1194 (726, 1980)	1188 (714, 1980)	1188 (732, 1980)
Menstruation regularity (no)	496 (16.6)	178 (17.9)	169 (17.0)	149 (15.0)	153 (15.4)	189 (19.0)	154 (15.5)
Family history of HG (yes)	813 (27.3)	256 (25.8)	301 (30.3)	256 (25.8)	264 (26.6)	284 (28.6)	265 (26.7)
Current smoker	53 (1.8)	9 (0.9)	21 (2.1)	23 (2.3)	17 (1.7)	12 (1.2)	24 (2.4)
Current alcohol drinker	107 (3.6)	41 (4.1)	32 (3.2)	34 (3.4)	28 (2.8)	38 (3.8)	41 (4.1)
Nutritional supplement usage (yes)	1788 (60.0)	602 (60.6)	597 (60.1)	589 (59.3)	603 (60.7)	593 (59.7)	592 (59.6)
Total energy intake (kcal/d)	1679 (1314, 2139)	1566 (1262, 2013)	1648 (1279, 2053)	1832 (1426, 2292)	1566 (1262, 2013)	1648 (1279, 2053)	1832 (1426, 2292)

Continuous variables were expressed as median (interquartile range) and categorical variables as n (%). Abbreviations: BMI: body mass index; CDAI: composite dietary antioxidant index; DAPS: dietary antioxidant potential score; HG: hyperemesis gravidarum; MET: metabolic equivalent.

**Table 2:** Associations between the CDAI or DAPS and HG risk (*n* = 2980) ^a^.

	Tertile 1	Tertile 2	Tertile 3	*p*-Trend	Per SD Increase
CDAI	(−1.60, −0.13)	(−0.13, 0.17)	(0.18, 1.35)	-	-
Case/total	118/993	86/993	37/994	-	-
Model 1	1.00 (reference)	0.70 (0.53, 0.94)	0.29 (0.20, 0.42)	<0.001	0.64 (0.72, 0.82)
Model 2	1.00 (reference.)	0.73 (0.55, 0.99)	0.31 (0.21, 0.47)	<0.001	0.75 (0.66, 0.85)
Model 3	1.00 (reference.)	0.66 (0.48, 0.90)	0.32 (0.21, 0.47)	<0.001	0.74 (0.64, 0.85)
DAPS	(−1.01, 1.21)	(1.22, 1.57)	(1.58, 3.50)	-	-
Case/total	112/994	85/993	44/993	-	-
Model 1	1.00 (reference.)	0.74 (0.55, 0.99)	0.37 (0.26, 0.52)	<0.001	0.53 (0.45, 0.64)
Model 2	1.00 (reference.)	0.75 (0.55, 1.00)	0.40 (0.28, 0.57)	<0.001	0.55 (0.46, 0.66)
Model 3	1.00 (reference.)	0.75 (0.55, 1.02)	0.41 (0.30, 0.60)	<0.001	0.56 (0.46, 0.67)

^a^ Values are odds ratios (95% confidence interval) estimated by binary logistic models. Model 1: unadjusted. Model 2: adjusted for age, pre-pregnancy body mass index, nutritional supplement usage, and total energy intake. Model 3 was further adjusted the gestational week, educational attainment, employment status, ethnicity, menstruation regularity, family history of HG, quality of life during pregnancy, physical activity, primigravida status, annual household income, current smoking status, and current alcohol consumption. Abbreviations: CDAI: composite dietary antioxidant index; CI: confidence interval; DAPS: dietary antioxidant potential score; HG: hyperemesis gravidarum; OR: odds ratio; SD: standard deviation.

## Data Availability

Due to restrictions on data protection, these materials can only be provided upon request. Requests for access to these materials should be sent via email to the corresponding author of this study.

## Appendix A

**Table A1:** Food groups for the dietary antioxidants potential score.

Food Groups	Weights ^a^	Factor Loadings ^b^
Positive associations		
Whole grains and mixed legumes	0.00271	0.28615
Fruits	0.00217	0.22074
Eggs	0.00173	0.04925
Fish	0.00163	0.03875
Dark green vegetables and white leafy vegetables	0.00109	0.06726
Yogurt	0.00081	0.05224
Milk	0.00032	0.04542
Water	0.00015	0.12943
Inverse associations		
Edible animal oils	−0.00694	−0.03727
Processed meat	−0.00192	−0.04321
Livestock meat	−0.00191	−0.13651
Poultry	−0.00095	−0.03594
Rice	−0.00047	−0.02659
Beverage	−0.00027	−0.02617
Explained variation in food groups, %	-	6.92
Explained variation in the composite dietary antioxidant index, %	-	29.51

^a^ The dietary antioxidant potential score is determined using regression coefficients acquired during the concluding phase of the stepwise linear regression models. These coefficients individually indicate the extent of contribution from each food group to the overall score. ^b^ Obtained through reduced rank regression analysis, where the dietary antioxidant index serves as the dependent variable in the modeling process.

**Table A2:** General characteristics of participants by HG status (*n* = 2980).

Characteristics	Non-HG (*n* = 2739)	HG (*n* = 241)
Age (years)	30.0 (28.0, 33.0)	30.0 (29.0, 33.0)
Gestational week (weeks)	12.0 (9.0, 12.7)	12.2 (11.3, 12.7)
Educational level (under college)	1516 (55.3)	147 (61.0)
Employment attainment (no)	534 (19.5)	56 (23.2)
Annual household income (<100,000 CNY)	1589 (58.0)	166 (68.9)
Ethnicity (Han)	2694 (98.4)	234 (97.1)
Primigravida (yes)	1315 (48.0)	79 (32.8)
Pre-pregnancy BMI (kg/m^2^)	21.2 (19.5, 23.3)	21.1 (19.7, 23.1)
Quality of life in pregnancy	8.0 (7.0, 9.0)	7.0 (5.0, 8.0)
Physical activity (MET-min/week))	1188 (738, 1980)	1086 (654, 1752)
Menstruation regularity (no)	463 (16.9)	33 (13.7)
Family history of hyperemesis gravidarum (yes)	719 (26.3)	94 (39.0)
Current smoker	47 (1.7)	6 (2.5)
Current alcohol drinker	97 (3.5)	10 (4.1)
Nutritional supplement usage (yes)	1641 (59.9)	147 (61.0)
Total energy intake (kcal/d)	1696.9 (1322.9, 2158.2)	1540.7 (1242.2, 1865.0)
Whole grains and mixed legumes (g/d)	120.8 (111.7, 147.9)	113.6 (102.9, 129.0)
Fruits (g/d)	171.7 (115.4, 261.2)	158.0 (100.5, 226.1)
Eggs (g/d)	25.5 (9.2, 57.0)	21.5 (7.2, 44.7)
Fish (g/d)	16.4 (9.4, 27.0)	13.4 (8.4, 21.0)
Dark green vegetables and white leafy vegetables (g/d)	251.9 (221.1, 297.3)	233.5 (209.3, 269.6)
Yogurt (g/d)	27.8 (15.1, 79.2)	25.8 (12.1, 77.2)
Milk (g/d)	117.5 (27.0, 208.6)	107.5 (17.0, 163.6)
Water (ml/d)	1350.0 (600.0, 2459.0)	1250.0 (500.0, 1250.0)
Edible animal oils (g/d)	1.1 (0.6, 3.3)	1.5 (0.6, 4.6)
Processed meat (g/d)	1.3 (1.0, 6.3)	1.7 (1.7, 6.7)
Livestock meat (g/d)	60.0 (30.4, 99.2)	65.6 (37.0, 107.9)
Poultry (g/d)	6.7 (6.7, 14.3)	14.3 (6.7, 25.3)
Rice (g/d)	160.0 (120.0, 175.1)	164.8 (125.0, 175.1)
Beverage (ml/d)	80.6 (57.1, 128.2)	81.4 (57.1, 131.7)
Vitamin A (RE)	552.3 (502.2, 593.1)	528.2 (496.5, 581.9)
Vitamin C (mg/d)	65.7 (63.1, 69.3)	63.0 (58.9, 66.6)
Vitamin E (mg/d)	10.4 (8.9, 11.9)	10.1 (9.2, 11.6)
Zinc (mg/d)	10.6 (8.9, 12.6)	9.7 (8.6, 11.3)
Selenium (mg/d)	78.2 (70.3, 86.6)	76.7 (70.5, 82.8)
Total carotenoids (mg/d)	6305.3 (5843.5, 6757.2)	6169.8 (5494.8, 6595.4)
CDAI	0.04 (−0.21, 0.25)	−0.12 (−0.29, 0.07)
DAPS	1.39 (1.13, 1.70)	1.23 (0.98, 1.48)

Continuous variables were expressed as median (interquartile range) and categorical variables as n (%). Abbreviations: BMI: body mass index; CDAI: composite dietary antioxidant index; DAPS: dietary antioxidant potential score; HG: hyperemesis gravidarum.

**Table A3:** Adjusted associations between the residual energy intake-adjusted CDAI and DAPS and the risk of hyperemesis gravidarum (n = 2980) ^1^.

	Tertile 1	Tertile 2	Tertile 3	*p*-Trend	Per SD Increase
Residual energy intake-adjusted CDAI					
Model 1	1.00 (reference)	0.75 (0.56, 1.01)	0.35 (0.23, 0.49)	<0.001	0.76 (0.67, 0.86)
Model 2	1.00 (reference)	0.15 (0.56, 1.01)	0.34 (0.23, 0.49)	<0.001	0.76 (0.67, 0.86)
Model 3	1.00 (reference)	0.60 (51, 0.94)	0.34 (0.23. 0.50)	<0.001	0.75 (0.66, 0.86)
Residual energy intake -adjusted DAPS					
Model 1	1.00 (reference)	0.73 (0.54, 0.99)	0.45 (0.32, 0.64)	<0.001	0.64 (0.55, 0.74)
Model 2	1.00 (reference)	0.73 (0.54, 0.99)	0.64 (0.55, 0.74)	<0.001	0.64 (0.55, 0.74)
Model 3	1.00 (reference)	0.70 (0.51, 0.96)	0.46 (0.32. 0.66)	<0.001	0.64 (0.54, 0.75)

^1^ Values are given as odds ratios and 95% confidence intervals within parentheses, the food group for the calculation of the dietary antioxidant potential score and six nutrients for the calculation of the CDAI were adjusted for total energy by using residual methods. Model 1: unadjusted. Model 2: adjusted for age, pre-pregnancy body mass index, nutritional supplement usage, and total energy intake. Model 3 was further adjusted the gestational week, educational attainment, employment status, ethnicity, menstruation regularity, family history of HG, quality of life during pregnancy, physical activity, primigravida status, annual household income, current smoking status, and current alcohol consumption. Abbreviation: CDAI: composite dietary antioxidant index; CI: confidence interval; DAPS: dietary antioxidant potential score; OR: odds ratio; SD: standard deviation.

**Table A4:** Adjusted associations between the CDAI and DAPS and the risk of hyperemesis gravidarum in the sensitive analysis ^a^.

	Tertile 1	Tertile 2	Tertile 3	*p*-Trend	Per SD Increase
CDAI ^1^ (*n* = 3074)	1.00 (reference)	0.74 (0.54, 0.99)	0.31 (0.21. 0.46)	<0.001	0.74 (0.64, 0.85)
CDAI ^2^ (*n* = 3364)	1.00 (reference)	0.74 (0.55, 0.99)	0.34 (0.24. 0.50)	<0.001	0.71 (0.62, 0.82)
DAPS ^1^ (*n* = 3074)	1.00 (reference)	0.72 (0.53, 0.98)	0.41 (0.28. 0.59)	<0.001	0.56 (0.47, 0.67)
DAPS ^2^ (*n* = 3364)	1.00 (reference)	0.78 (0.58,1.04)	0.44 (0.31. 0.62)	<0.001	0.64 (0.56, 0.75)

^a^ Values are given as odds ratios and 95% confidence intervals within parentheses. ^1^ Included the participants with implausible total energy intake estimates of below 500 kcal/day or above 3500 kcal/day. ^2^ Included the participants where multiple imputation methods were employed for missing covariates. Multivariable logistics models adjusted for age, week of gestation, pre-pregnancy body mass index, use of the nutritional supplement, educational attainment, employment status, ethnicity, pre-pregnancy body mass index, regularity of menstruation, family history of hyperemesis gravidarum, quality of life in pregnancy, physical activity, primigravida, annual household income, current smoker, ethnicity and alcohol drinking status. Abbreviation: CDAI: composite dietary antioxidant index; CI: confidence interval; DAPS: dietary antioxidant potential score; OR: odds ratio; SD: standard deviation.

## References

[1] Einarson T.R., Piwko C., Koren G. (2013). Quantifying the global rates of nausea and vomiting of pregnancy: A meta analysis. J. Popul. Ther. Clin. Pharmacol..

[2] Fejzo M.S., Trovik J., Grooten I.J., Sridharan K., Roseboom T.J., Vikanes Å., Painter R.C., Mullin P.M. (2019). Nausea and vomiting of pregnancy and hyperemesis gravidarum. Nat. Rev. Dis. Primers.

[3] Deruelle P., Sentilhes L., Ghesquière L., Desbrière R., Ducarme G., Attali L., Jarnoux A., Artzner F., Tranchant A., Schmitz T. (2024). Nausea and vomiting in pregnancy. Rev. Prat..

[4] Nijsten K., Jansen L.A.W., Limpens J., Finken M.J.J., Koot M.H., Grooten I.J., Roseboom T.J., Painter R.C. (2022). Long-term health outcomes of children born to mothers with hyperemesis gravidarum: A systematic review and meta-analysis. Am. J. Obstet. Gynecol..

[5] Fiaschi L., Nelson-Piercy C., Gibson J., Szatkowski L., Tata L.J. (2018). Adverse Maternal and Birth Outcomes in Women Admitted to Hospital for Hyperemesis Gravidarum: A Population-Based Cohort Study. Paediatr. Perinat. Epidemiol..

[6] Şimşek Y., Şimşek G., Bayar Muluk N., Arıkan O.K. (2021). Olfactory dysfunction and oxidative stress in pregnant women with hyperemesis gravidarum. Arch. Gynecol. Obstet..

[7] Fisher J.J., Bartho L.A., Perkins A.V., Holland O.J. (2020). Placental mitochondria and reactive oxygen species in the physiology and pathophysiology of pregnancy. Clin. Exp. Pharmacol. Physiol..

[8] Ballester P., Cerdá B., Arcusa R., Marhuenda J., Yamedjeu K., Zafrilla P. (2022). Effect of Ginger on Inflammatory Diseases. Molecules.

[9] Tan P.C., Ramasandran G., Sethi N., Razali N., Hamdan M., Kamarudin M. (2023). Watermelon and dietary advice compared to dietary advice alone following hospitalization for hyperemesis gravidarum: A randomized controlled trial. BMC Pregnancy Childbirth.

[10] Sharifzadeh F., Kashanian M., Koohpayehzadeh J., Rezaian F., Sheikhansari N., Eshraghi N. (2018). A comparison between the effects of ginger, pyridoxine (vitamin B6) and placebo for the treatment of the first trimester nausea and vomiting of pregnancy (NVP). J. Matern. Fetal Neonatal Med..

[11] Celik F., Guzel A.I., Kuyumcuoglu U., Celik Y. (2011). Dietary antioxidant levels in hyperemesis gravidarum: A case control study. Ginekol. Pol..

[12] Cheng W., Li L., Long Z., Ma X., Chen F., Ma L., Zhang S., Lin J. (2023). Association between Dietary Patterns and the Risk of Hyperemesis Gravidarum. Nutrients.

[13] Zhi S., Zhang L., Cheng W., Jin Y., Long Z., Gu W., Ma L., Zhang S., Lin J. (2024). Association between Dietary Inflammatory Index and Hyperemesis Gravidarum. Nutrients.

[14] Yu Y.C., Paragomi P., Wang R., Jin A., Schoen R.E., Sheng L.T., Pan A., Koh W.P., Yuan J.M., Luu H.N. (2022). Composite dietary antioxidant index and the risk of colorectal cancer: Findings from the Singapore Chinese Health Study. Int. J. Cancer.

[15] Wang M., Huang Z.H., Zhu Y.H., He P., Fan Q.L. (2023). Association between the composite dietary antioxidant index and chronic kidney disease: Evidence from NHANES 2011–2018. Food Funct..

[16] Wright M.E., Mayne S.T., Stolzenberg-Solomon R.Z., Li Z., Pietinen P., Taylor P.R., Virtamo J., Albanes D. (2004). Development of a comprehensive dietary antioxidant index and application to lung cancer risk in a cohort of male smokers. Am. J. Epidemiol..

[17] Zhao J., Li Z., Gao Q., Zhao H., Chen S., Huang L., Wang W., Wang T. (2021). A review of statistical methods for dietary pattern analysis. Nutr. J..

[18] Zhang S., Meng G., Zhang Q., Liu L., Wu H., Gu Y., Wang Y., Zhang T., Wang X., Zhang J. (2022). Inflammatory potential of diet and risk of nonalcoholic fatty liver disease: A prospective cohort study. Eur. J. Clin. Nutr..

[19] Piernas C., Gao M., Jebb S.A. (2022). Dietary patterns derived by reduced rank regression and non-communicable disease risk. Proc. Nutr. Soc..

[20] Frankenfeld C.L. (2021). Reduced Rank Regression: Illustration of an Important Tool in the Evaluation of Dietary Patterns and Chronic Disease Risk. J. Nutr..

[21] Skulsky S.L., Koutoukidis D.A., Carter J.L., Piernas C., Jebb S.A., Gao M., Astbury N.M. (2024). Associations between Dietary Patterns and Incident Colorectal Cancer in 114,443 Individuals from the UK Biobank: A Prospective Cohort Study. Cancer Epidemiol. Biomarkers Prev..

[22] Fan Y., Li Z., Shi J., Liu S., Li L., Ding L., Zhao J., Pan Y., Lei H., He T. (2024). The association between prepregnancy dietary fatty acids and risk of gestational diabetes mellitus: A prospective cohort study. Clin. Nutr..

[23] Cheng Y., Dibley M.J., Zhang X., Zeng L., Yan H. (2009). Assessment of dietary intake among pregnant women in a rural area of western China. BMC Public Health.

[24] Cheng Y., Yan H., Dibley M.J., Shen Y., Li Q., Zeng L. (2008). Validity and reproducibility of a semi-quantitative food frequency questionnaire for use among pregnant women in rural China. Asia Pac. J. Clin. Nutr..

[25] Xu Q., Qian X., Sun F., Liu H., Dou Z., Zhang J. (2023). Independent and joint associations of dietary antioxidant intake with risk of post-stroke depression and all-cause mortality. J. Affect. Disord..

[26] Yang J., Na X., Li Z., Zhao A. (2024). Modification Role of Dietary Antioxidants in the Association of High Red Meat Intake and Lung Cancer Risk: Evidence from a Cancer Screening Trial. Antioxidants.

[27] Koren G., Boskovic R., Hard M., Maltepe C., Navioz Y., Einarson A. (2002). Motherisk-PUQE (pregnancy-unique quantification of emesis and nausea) scoring system for nausea and vomiting of pregnancy. Am. J. Obstet. Gynecol..

[28] Jansen L.A.W., Shaw V., Grooten I.J., Koot M.H., Dean C.R., Painter R.C. (2024). Diagnosis and treatment of hyperemesis gravidarum. Cmaj.

[29] Jansen L.A.W., Koot M.H., Van’t Hooft J., Dean C.R., Bossuyt P.M.M., Ganzevoort W., Gauw N., Van der Goes B.Y., Rodenburg J., Roseboom T.J. (2021). The windsor definition for hyperemesis gravidarum: A multistakeholder international consensus definition. Eur. J. Obstet. Gynecol. Reprod. Biol..

[30] Koot M.H., Grooten I.J., Post J., Bais J.M.J., Ris-Stalpers C., Naaktgeboren C.A., Niemeijer M.N., Bremer H.A., van der Ham D.P., Heidema W.M. (2020). Ketonuria is not associated with hyperemesis gravidarum disease severity. Eur. J. Obstet. Gynecol. Reprod. Biol..

[31] Tennant P.W.G., Murray E.J., Arnold K.F., Berrie L., Fox M.P., Gadd S.C., Harrison W.J., Keeble C., Ranker L.R., Textor J. (2021). Use of directed acyclic graphs (DAGs) to identify confounders in applied health research: Review and recommendations. Int. J. Epidemiol..

[32] Xiao X., Qin Z., Lv X., Dai Y., Ciren Z., Yangla Y., Zeng P., Ma Y., Li X., Wang L. (2021). Dietary patterns and cardiometabolic risks in diverse less-developed ethnic minority regions: Results from the China Multi-Ethnic Cohort (CMEC) Study. Lancet Reg. Health West. Pac..

[33] Cheng J., Sun J., Yao K., Xu M., Cao Y. (2022). A variable selection method based on mutual information and variance inflation factor. Spectrochim. Acta A Mol. Biomol. Spectrosc..

[34] Willett W.C., Howe G.R., Kushi L.H. (1997). Adjustment for total energy intake in epidemiologic studies. Am. J. Clin. Nutr..

[35] Tomova G.D., Arnold K.F., Gilthorpe M.S., Tennant P.W.G. (2022). Adjustment for energy intake in nutritional research: A causal inference perspective. Am. J. Clin. Nutr..

[36] Beesley L.J., Bondarenko I., Elliot M.R., Kurian A.W., Katz S.J., Taylor J.M. (2021). Multiple imputation with missing data indicators. Stat. Methods Med. Res..

[37] Alharbi K.S., Nadeem M.S., Afzal O., Alzarea S.I., Altamimi A.S.A., Almalki W.H., Mubeen B., Iftikhar S., Shah L., Kazmi I. (2022). Gingerol, a Natural Antioxidant, Attenuates Hyperglycemia and Downstream Complications. Metabolites.

[38] Tiran D. (2012). Ginger to reduce nausea and vomiting during pregnancy: Evidence of effectiveness is not the same as proof of safety. Complement. Ther. Clin. Pract..

[39] Burton G.J., Jauniaux E. (2011). Oxidative stress. Best. Pract. Res. Clin. Obstet. Gynaecol..

[40] Poston L., Raijmakers M.T. (2004). Trophoblast oxidative stress, antioxidants and pregnancy outcome—A review. Placenta.

[41] Pingitore A., Lima G.P., Mastorci F., Quinones A., Iervasi G., Vassalle C. (2015). Exercise and oxidative stress: Potential effects of antioxidant dietary strategies in sports. Nutrition.

[42] Wang X., Quinn P.J. (1999). Vitamin E and its function in membranes. Prog. Lipid Res..

[43] Sikiru A.B., Arangasamy A., Alemede I.C., Guvvala P.R., Egena S.S.A., Ippala J.R., Bhatta R. (2019). Chlorella vulgaris supplementation effects on performances, oxidative stress and antioxidant genes expression in liver and ovaries of New Zealand White rabbits. Heliyon.

[44] Li Y., Matsuzaki N., Masuhiro K., Kameda T., Taniguchi T., Saji F., Yone K., Tanizawa O. (1992). Trophoblast-derived tumor necrosis factor-alpha induces release of human chorionic gonadotropin using interleukin-6 (IL-6) and IL-6-receptor-dependent system in the normal human trophoblasts. J. Clin. Endocrinol. Metab..

[45] Nestler J.E. (1993). Interleukin-1 stimulates the aromatase activity of human placental cytotrophoblasts. Endocrinology.

[46] Cunningham A.S., Muneyyirci-Delale O. (2009). The association between primary dysmenorrhea and hyperemesis gravidarum. Med. Hypotheses.

[47] Luu H.N., Wen W., Li H., Dai Q., Yang G., Cai Q., Xiang Y.B., Gao Y.T., Zheng W., Shu X.O. (2015). Are dietary antioxidant intake indices correlated to oxidative stress and inflammatory marker levels?. Antioxid. Redox Signal.

[48] Trebble T., Arden N.K., Stroud M.A., Wootton S.A., Burdge G.C., Miles E.A., Ballinger A.B., Thompson R.L., Calder P.C. (2003). Inhibition of tumour necrosis factor-alpha and interleukin 6 production by mononuclear cells following dietary fish-oil supplementation in healthy men and response to antioxidant co-supplementation. Br. J. Nutr..

[49] Novoselova E.G., Lunin S.M., Novoselova T.V., Khrenov M.O., Glushkova O.V., Avkhacheva N.V., Safronova V.G., Fesenko E.E. (2009). Naturally occurring antioxidant nutrients reduce inflammatory response in mice. Eur. J. Pharmacol..

[50] Plunkett B.A., Callister R., Watson T.A., Garg M.L. (2010). Dietary antioxidant restriction affects the inflammatory response in athletes. Br. J. Nutr..

[51] Vahid F., Rahmani D., Davoodi S.H. (2021). The correlation between serum inflammatory, antioxidant, glucose handling biomarkers, and Dietary Antioxidant Index (DAI) and the role of DAI in obesity/overweight causation: Population-based case-control study. Int. J. Obes..

[52] DiPalma J.R., Ritchie D.M. (1977). Vitamin toxicity. Annu. Rev. Pharmacol. Toxicol..

[53] Thorlton J., Ahmed A., Colby D.A. (2016). Energy Drinks: Implications for the Breastfeeding Mother. MCN Am. J. Matern. Child. Nurs..

[54] Cucó G., Fernández-Ballart J., Sala J., Viladrich C., Iranzo R., Vila J., Arija V. (2006). Dietary patterns and associated lifestyles in preconception, pregnancy and postpartum. Eur. J. Clin. Nutr..

